# Graphene-Ionic Liquid Thin Film Nanolubricant

**DOI:** 10.3390/nano10030535

**Published:** 2020-03-17

**Authors:** María-Dolores Avilés, Ramón Pamies, José Sanes, María-Dolores Bermúdez

**Affiliations:** Group of Materials Science and Metallurgical Engineering, Technical University of Cartagena, Campus Muralla del Mar, 30202 Cartagena, Spain; mdolores.aviles@upct.es (M.-D.A.); ramon.pamies@upct.es (R.P.); pepe.sanes@upct.es (J.S.)

**Keywords:** graphene, ionic liquid, thin film, spin coating

## Abstract

Graphene (0.5 wt.%) was dispersed in the hydrophobic room-temperature ionic liquid 1-octyl-3-methylimidazolium bis(trifluoromethanesulfonyl) imide (IL) to obtain a new non-Newtonian (IL + G) nanolubricant. Thin layers of IL and (IL + G) lubricants were deposited on stainless steel disks by spin coating. The tribological performance of the new thin layers was compared with those of full fluid lubricants. Friction coefficients for neat IL were independent of lubricant film thickness. In contrast, for (IL + G) the reduction of film thickness not only afforded 40% reduction of the friction coefficient, but also prevented wear and surface damage. Results of surface profilometry, scanning and transmission electron microscopy (SEM and TEM), energy dispersive analysis (EDX), X-ray photoelectron spectroscopy (XPS) and Raman spectroscopy were discussed.

## 1. Introduction

The excellent performance of ionic liquids (ILs) as lubricants has been a subject of study for the last two decades [1,2,3,4]. Alkylimidazolium salts with fluorinated anions were the first to be investigated and feature among those ILs that have received deeper attention. Two main structural features were found to influence their properties, namely alkyl chain length and anion composition. As has been previously described [5], the load carrying ability of ILs in metal–metal and metal–ceramic contacts is enhanced by increasing alkyl chain lengths. An octyl-methyl-imidazolium cation was selected following previous results for (OMIM]BF_4_ + graphene dispersions and because ionic liquids with longer alkyl chain cations could be solid at room temperature, while the hydrophobic nature of bis(trifluoromethanesulfonyl) imide ([TFSI]) was one of the factors considered for its selection. Although hydrophobicity and stability towards hydrolysis are two factors that are not necessarily correlated, tetrafluoroborate derivatives with short alkyl lateral groups may cause metal tribocorrosion and subsequent formation of metal fluorides when sliding under atmospheric conditions [6,7].

Among the wide spectrum of potential applications of graphene and graphene derivatives, such as graphene oxide (GO), there has been a recent interest in their use as surface modifiers and lubricant additives [7,8]. The chemical nature of ILs is an effective instrument for the surface modification and/or functionalization of nanoparticles and carbon nanophases [9,10,11,12,13,14,15,16,17,18,19,20,21,22,23,24]. IL–graphene interactions may improve the dispersibility of the nanophases by inhibiting agglomeration. The most common strategy for the development of new graphene–IL nanophases for tribological applications is that of chemical functionalization. Khare et al. [19] were the first to combine graphene with an ionic liquid, the high viscosity 1-butyl-3-methylimidazolium iodide ([BMIM]I), in a new hybrid IL–graphene nanolubricant. IL-functionalized multilayer graphene was used as additive in IL to reduce friction and wear in steel–steel contacts [20].

The excellent tribological properties of lubricant additives of GO modified by ILs are attributed to the formation of an IL-containing graphene rich tribofilm on the sliding surfaces, which prevents direct asperity contact. Among the ILs studied was 1-hexyl-3-methylimidazolium bis(trifluoromethylsulfonyl) imide ([HMIM][TFSI]) [21]. For graphene covalently attached to imidazolium rings, graphene–IL surface layers were also found on wear tracks together with tribochemical reaction products [22].

Hybrid graphene–IL nanolubricants have also been used to generate thin surface lubricating films on silicon substrates by electrostatic adsorption [18] or by dip-coating [23] methods. It was concluded that the formation of a continuous IL–G surface film was necessary to decrease wear.

The synergistic effect of a physical adsorption film and a tribo-chemical reaction film on the sliding surfaces were proposed to explain the friction- and wear-reducing performance of the new IL-modified multilayer graphene dispersed in IL [24].

Our research group has recently developed a simple spin coating procedure to generate thin surface lubricant layers [13] or surface coatings [25]. In the present work, we compare the tribological performance of neat IL and IL–graphene lubricants both under thick film and thin film lubrication conditions.

The selected IL, 1-octyl-3-methylimidazolium bis(trifluoromethanesulfonyl) imide ([OMIM][TFSI]), combines a long alkyl chain with a hydrophobic anion. The interactions of [OMIM][TFSI] with the graphite surface has been the object of recent studies [26,27]. It was found that [OMIM] + cations adopt a parallel orientation on a highly-oriented pyrolytic graphite surface, with higher adsorption energy than other alkylmethylimidazolium cations with shorter alkyl chains. This was attributed to stronger van der Waals interactions as the number of carbon atoms in the lateral chain increases. This parallel orientation could favor the shearing of graphene sheets at the sliding contact, thus enhancing the lubricating performance of a hybrid graphene–[OMIM][TFSI] lubricant [28].

Once the nature of the IL was selected, the next critical factor was graphene concentration. Previous studies [29] have shown that low concentrations (with respect to 1 wt.%) have no effect on the lubricating performance, while high concentrations result in the formation of large aggregates that can inhibit sliding at the interface. An intermediate concentration value of 0.5 wt.% was selected to develop the new (IL + G) lubricant studied in the present work.

Another relevant factor in the selection of the 0.5 wt.% graphene proportion is the recently described [30] influence of graphene proportion on the variation of viscosity with temperature for graphene dispersions in ionic liquids.

For 1-ethyl-3-methylimidazolium ILs, dispersions with graphene contents higher than 0.5 wt.%, show unexpected rheological behavior, with linearly increasing viscosity under increasing temperature conditions. However, the viscosity of IL + 0.5% graphene becomes asymptotic with temperature and remains constant from 112 °C. Moreover, the dispersion with 0.5 wt.% graphene content showed the best tribological performance.

## 2. Materials and Methods

A commercial form of graphene (1–10 layers; thickness 0.55–3.74 nm; size 0.5–3 μm; purity > 99%; Iolitec, Heilbronn, Germany) was used. The ionic liquid 1-octyl-3-methylimidazolium bis(trifluoromethane sulfonyl) imide (IL) (purity 99.5%) was purchased from Solvionic (Toulouse, France).

**Figure 1:**
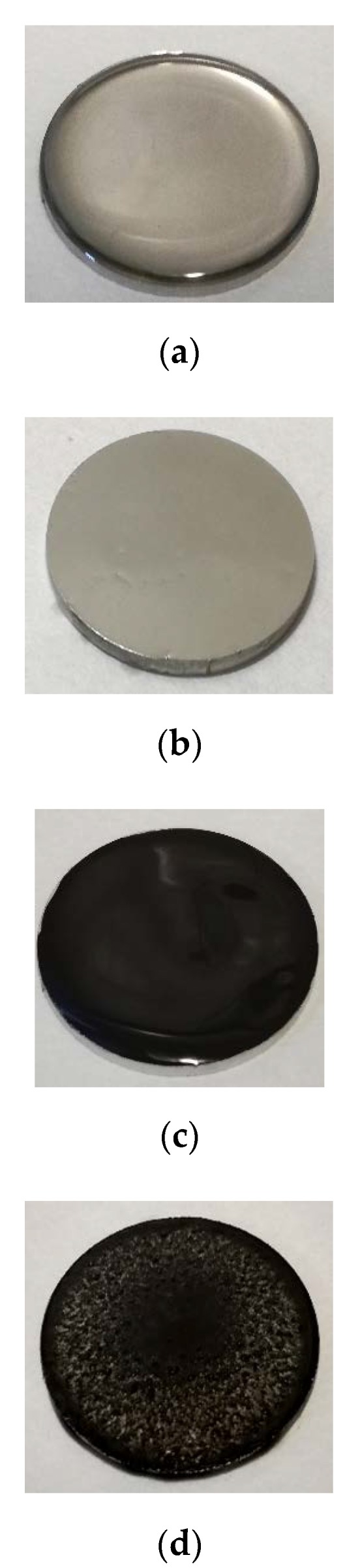
Photographs of AISI 316L disks covered with the lubricants before the tests: (**a**) IL; (**b**) IL spin coated thin film; (**c**) IL + G; (**d**) IL + G spin coated thin film.

The previously described [13,14] procedure of mechanical milling and sonication was used to obtain the (IL + G) dispersion. IL and (IL + G) thin layers were obtained by spin coating (POLOS TM, SPS-Europe B.V., Amsterdam, The Netherlands) at 1000 rpm for 30 s. Steel surfaces covered with the different lubricants are shown in Figure 1a–d. For full-fluid lubrication, the steel surface was completely covered by a volume of lubricant of 0.2 mL. In the case of IL, lubricant thickness was reduced by approximately 90%, from 340 µm (Figure 1a) to 34 μm (Figure 1b) by spin coating. For (IL + G), film thickness decreased from 470 µm (Figure 1c) to 128 μm (Figure 1d). Film thickness values are approximate and were estimated by a gravimetric method from the lubricant mass deposited on the total circular area of the steel disk.

AISI 316L stainless steel disks and sapphire balls were used for pin-on-disk tribological tests, under the conditions shown in Table 1, and previously described for sapphire–steel IL lubrication.

Rheological determinations were made by means of a double plate (plate diameter 40 mm; distance between plates 1000 µm) rheometer (AR-G2; TA Instruments, Lubbock, TX, USA), with a Peltier temperature control system. Tribological tests were performed in a pin-on-disk TRB tribometer (Anton Paar GmbH, Masó Analítica, Spain). All tests were repeated at least 3 times. Friction coefficients were continuously recorded during the tests. Test materials were cleaned with n-hexane and dried in hot air. Surface roughness, surface topography and wear rates were determined from profilometry measurements (Talysurf CLI; Taylor Hobson, Chicago, IL, USA). Wear debris were washed with acetone, centrifuged at 4400 rpm for 3 min and dried at 60 °C for 24 h, before microscopy observations. An S3500N (Hitachi, Japan) scanning electron microscope (SEM) was used to obtain micrographs and energy dispersive (EDX) spectra. The high resolution JEOL JEM 2100 transmission electron microscope (TEM) was used to study wear debris. XPS analysis was obtained with a K-Alpha Thermo-Scientific equipment, with 0.1 eV precision for binding energy values. Raman spectra were recorded with a 514 nm laser using a Renishaw inVia and a Leica microscope.

## 3. Results and Discussion

### 3.1. Rheological Behavior

**Figure 2:**
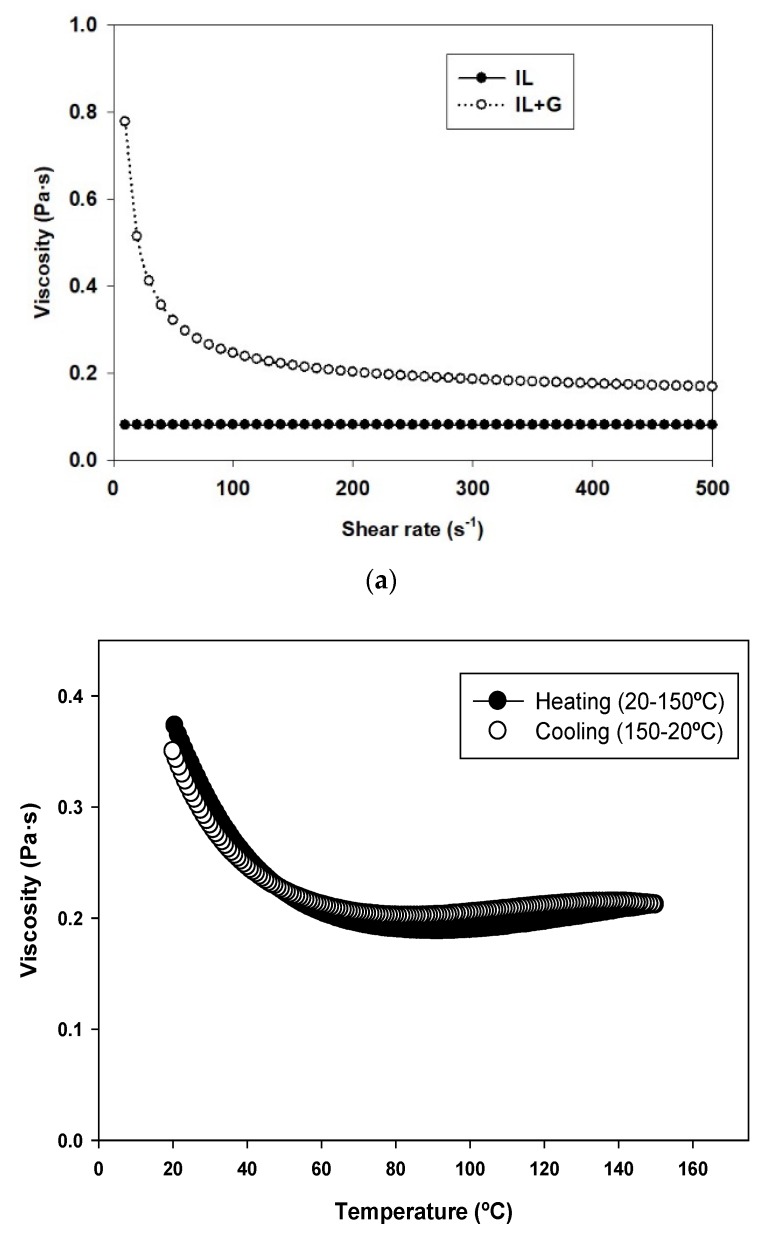
(**a**) Viscosity vs. shear rate at 25 °C; (**b**) viscosity vs. temperature for (IL + G) dispersion.

Figure 2a shows the influence of the dispersion of graphene on the rheological behavior of the ionic liquid. While neat IL shows the characteristics of a Newtonian fluid [29], with a constant viscosity value of 0.08 Pa·s under increasing shear rate, the addition of 0.5 wt.% graphene increased viscosity and yielded a non-Newtonian behavior, with decreasing viscosity values, from 0.78 to 0.17 Pa·s, as shear rate increased from 0 to 500 s^−1^. The presence of graphene strongly inhibited the mobility of IL molecules, particularly under low shear.

We previously described [30] the unusual temperature effect on the rheology of 1-ethyl-3-methylimidazolium ([EMIM]) ILs, with dicyanamide ([DCA]) or [TFSI] anions, containing variable concentrations of dispersed graphene. The viscosity of [EMIM][DCA] + 0.5 wt.% graphene and [EMIM][TFSI] + 0.5 wt.% graphene reached an asymptotic behavior with increasing temperature, while for 0.75 wt.% and 1.0 wt.% graphene, the viscosity values increased with temperature increase.

Figure 2b shows that IL + G, containing 0.5 wt.% graphene dispersed in [OMIM][TFSI], presented the expected viscosity decrease with temperature increase and the corresponding viscosity increase with temperature decrease, from 0.37 Pa·s at 20 °C to 0.19 Pa·s at 100 °C. However, from 100 to 150 °C viscosity values were constant or even showed a very slight increase to 0.21 Pa·s at 150 °C. This rheological behavior is similar to that of 1-ethyl-3-methylimidazolium ILs with 0.5 wt.% graphene [29] and could be tentatively attributed to the mobility hindrance of IL molecules by increasing interaction with graphene surface. These new IL–graphene dispersions open a new line of lubricants with controlled viscosity under increasing temperature conditions.

### 3.2. Friction Coefficients and Wear Rates

**Figure 3:**
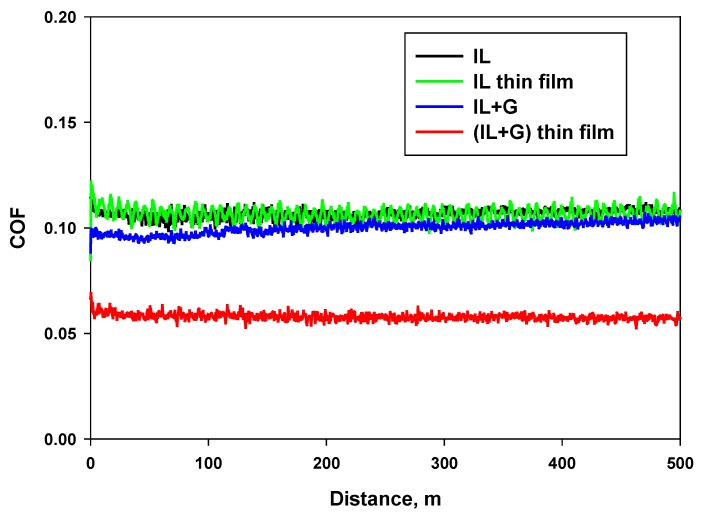
Coefficient of friction vs. sliding distance records for each lubricant.

Tribological results are shown in Table 2. The coefficient of friction obtained for IL lubricant (0.10) was not reduced by the addition of graphene (IL + G) nor by reduction of film thickness (IL thin film). In contrast, for (IL + G) and (IL + G) thin film, film thickness reduction produced a 40% reduction of friction coefficient. All lubricants showed constant friction records with sliding distance (Figure 3), without running-in periods, reaching steady-state lubrication regimes from the start of the sliding. One of the reasons for this constant friction coefficient with sliding distance could be the high thermal stability of the lubricants.

**Figure 4:**
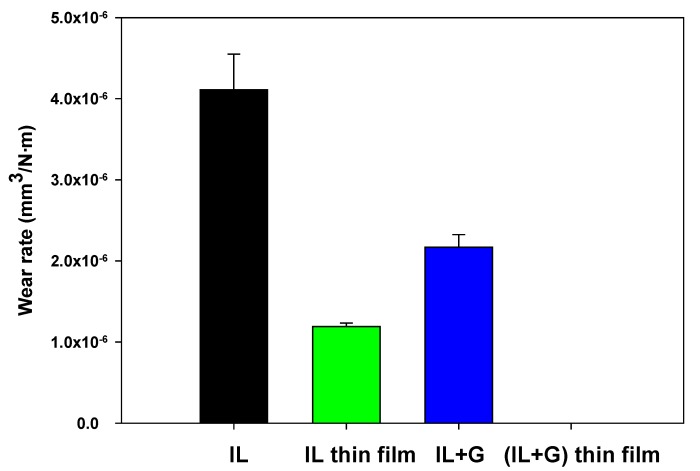
Wear rates of AISI 316L disks.

A 54% wear rate reduction (Table 2; Figure 4) for AISI 316L steel disks was obtained by the addition of graphene (IL + G) to neat IL. A higher reduction of 70% was obtained by IL film thickness reduction (IL thin film). The combination of both factors, namely the addition of graphene and film thickness reduction in (IL + G) thin film, reduced wear and surface damage to an unmeasurable scale, which is discussed in Section 3.3.

### 3.3. Surface Analysis and Wear Mechanism

**Figure 5:**
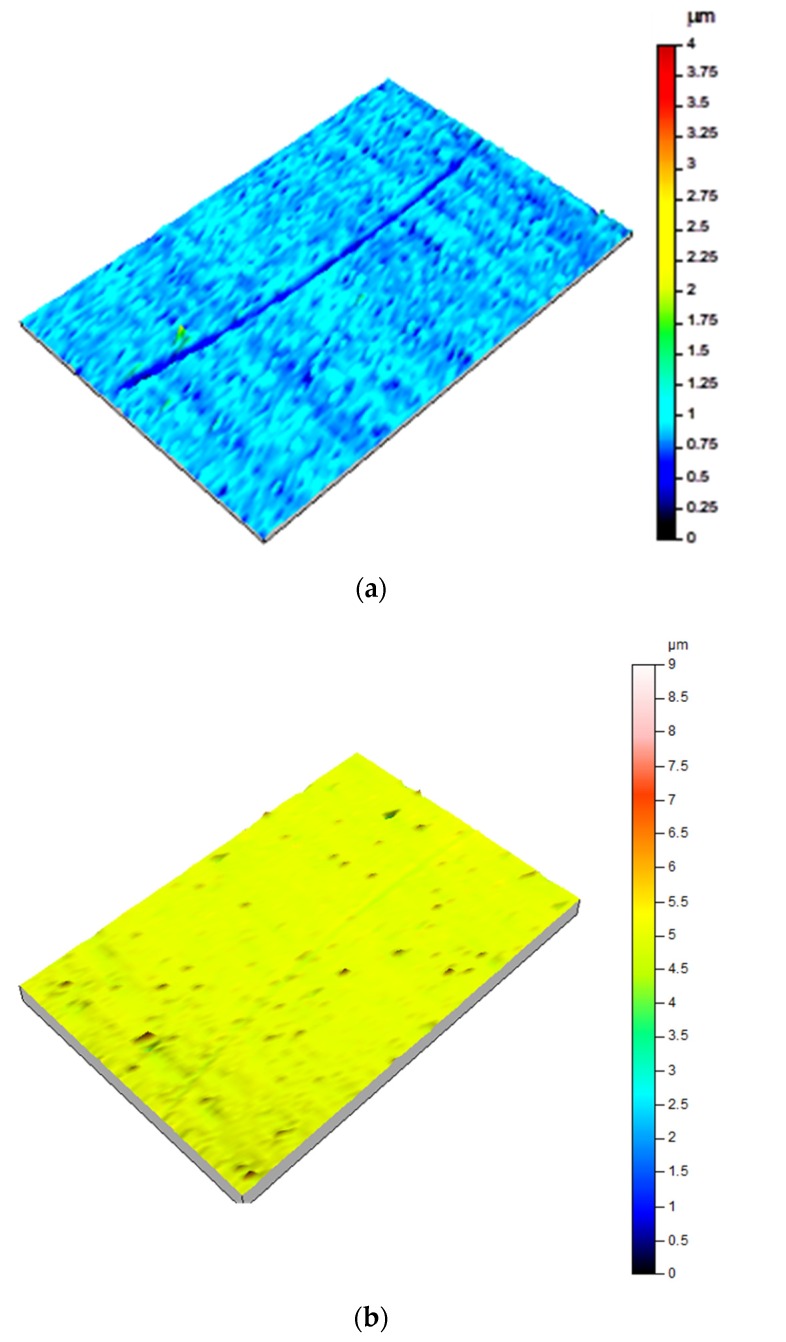
Surface topography profiles of AISI 316L disks after the tribological tests: (**a**) IL; (**b**) IL thin film.

**Figure 6:**
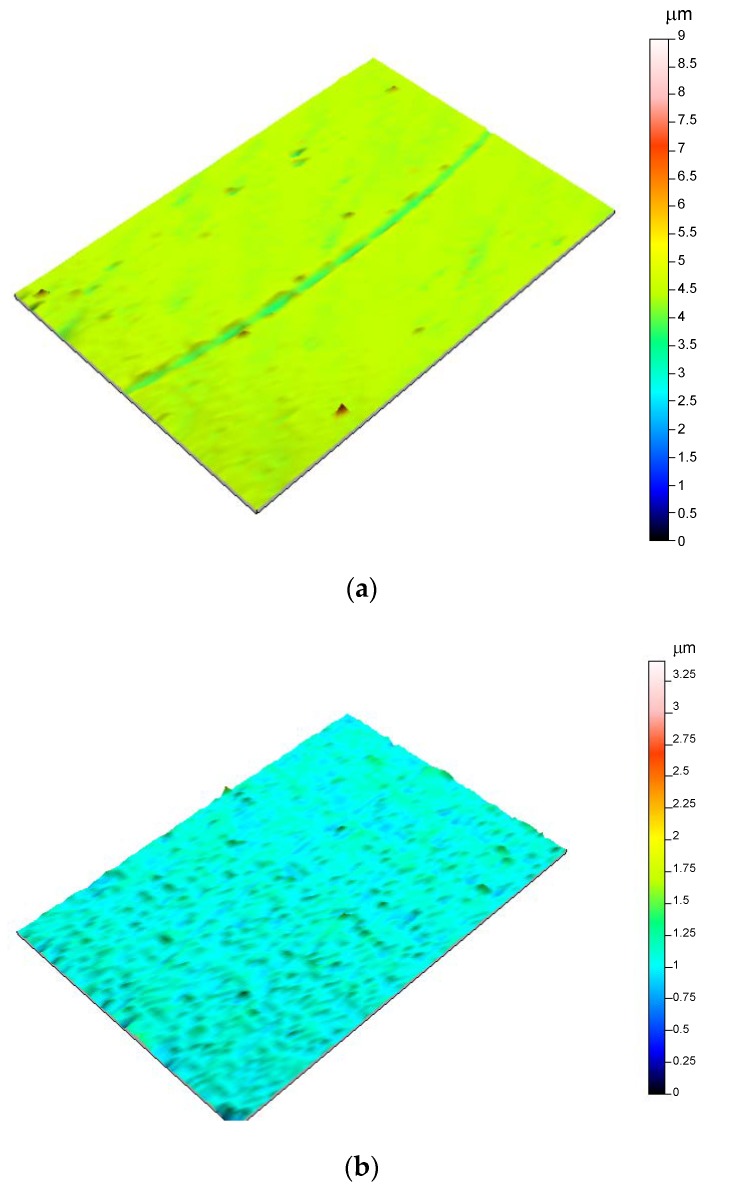
Surface topography profiles of AISI 316L disks after the tribological tests: (**a**) IL + G; (**b**) (IL + G) thin film.

**Figure 7:**
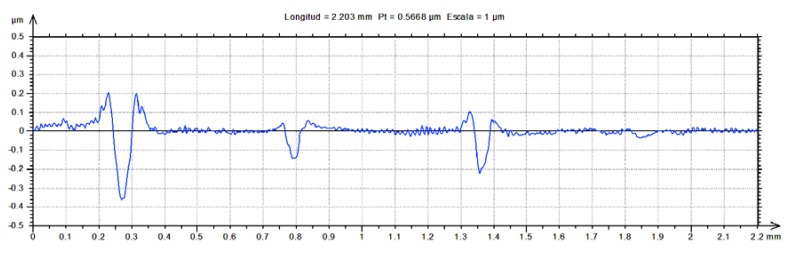
Cross section profiles on AISI 316L disks after the tribological tests with the four lubricants.

**Figure 8:**
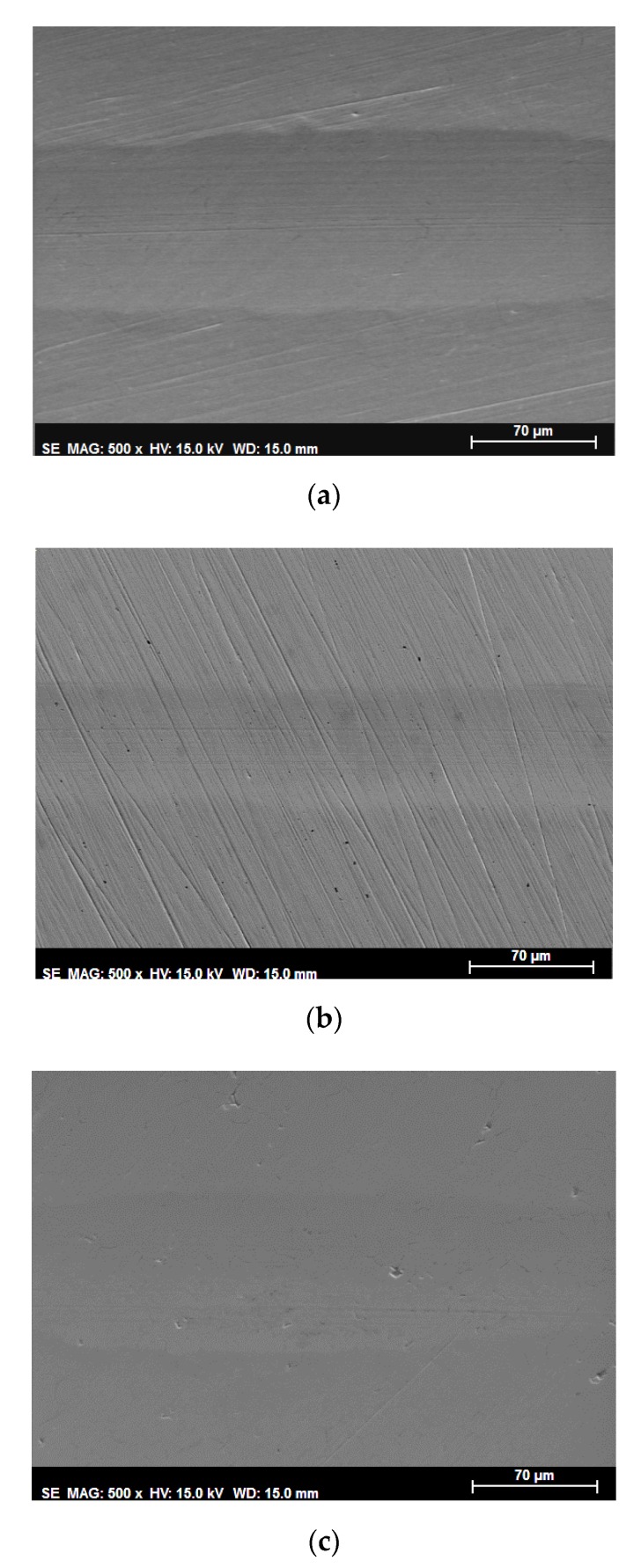

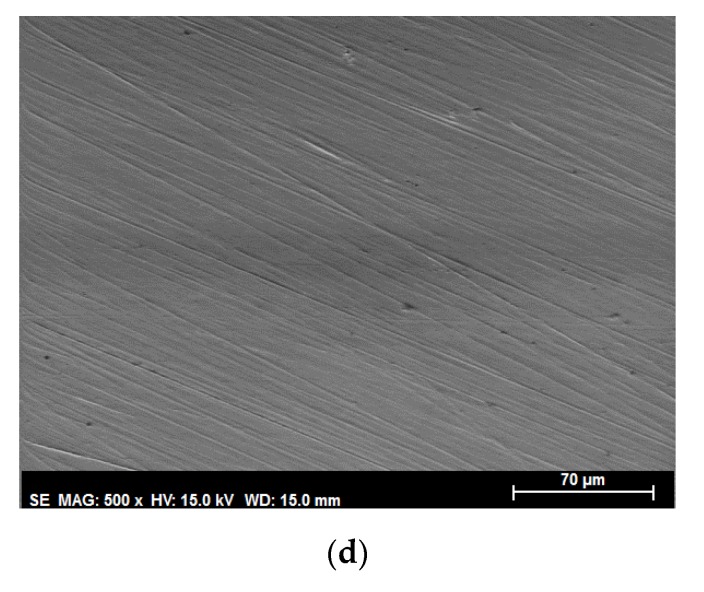
SEM micrographs of wear tracks on AISI 316L after the tribological tests: (**a**) IL; (**b**) IL thin film; (**c**) IL + G; (**d**) IL + G thin film.

**Figure 9:**
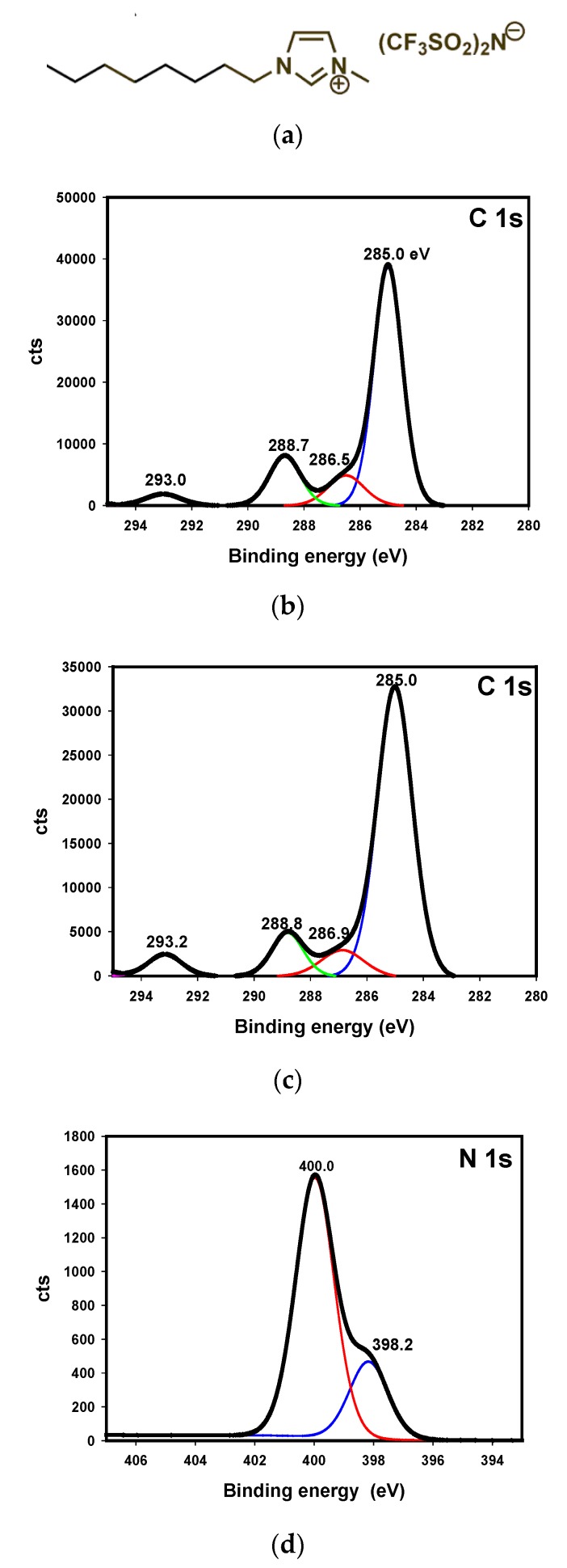

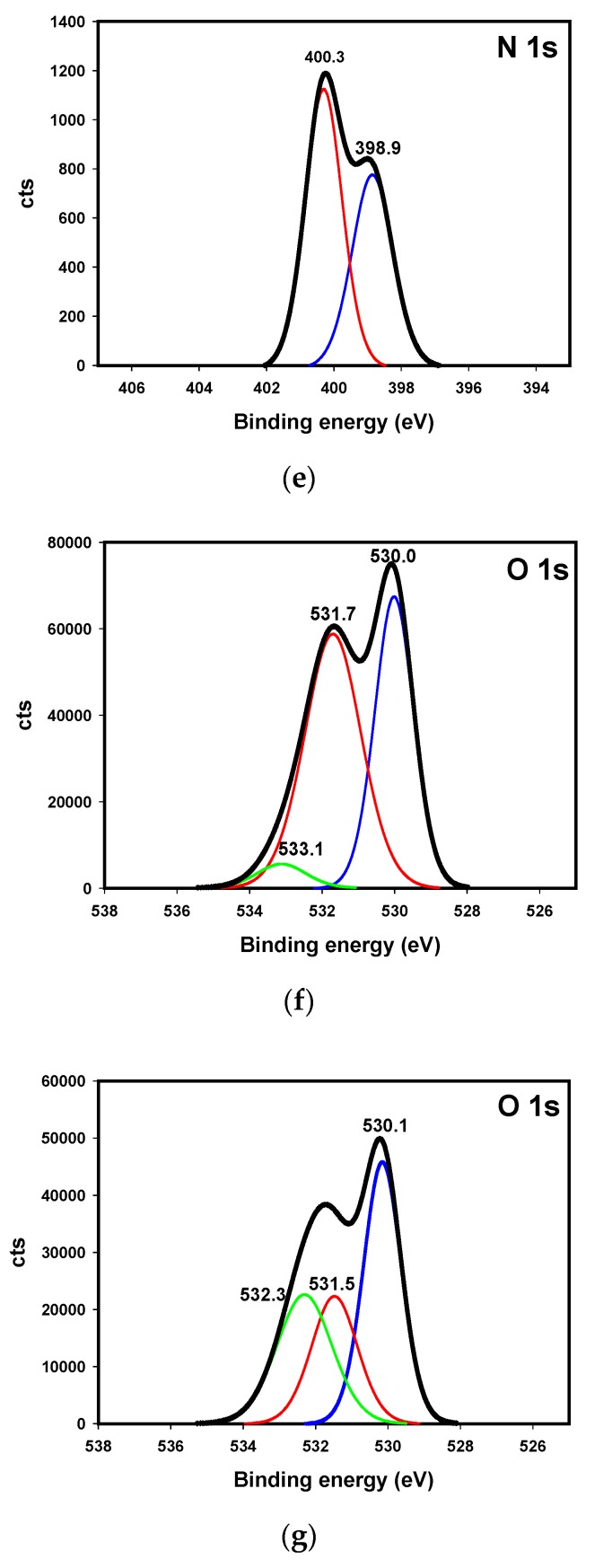
(**a**) Chemical formula of [OMIM][TFSI]. C1s binding energies: (**b**) outside the wear track; (**c**) inside the wear track. N1s binding energies: (**d**) outside the wear track; (**e**) inside the wear track. O1s binding energies: (**f**) outside the wear track; (**g**) inside the wear track.

**Figure 10:**
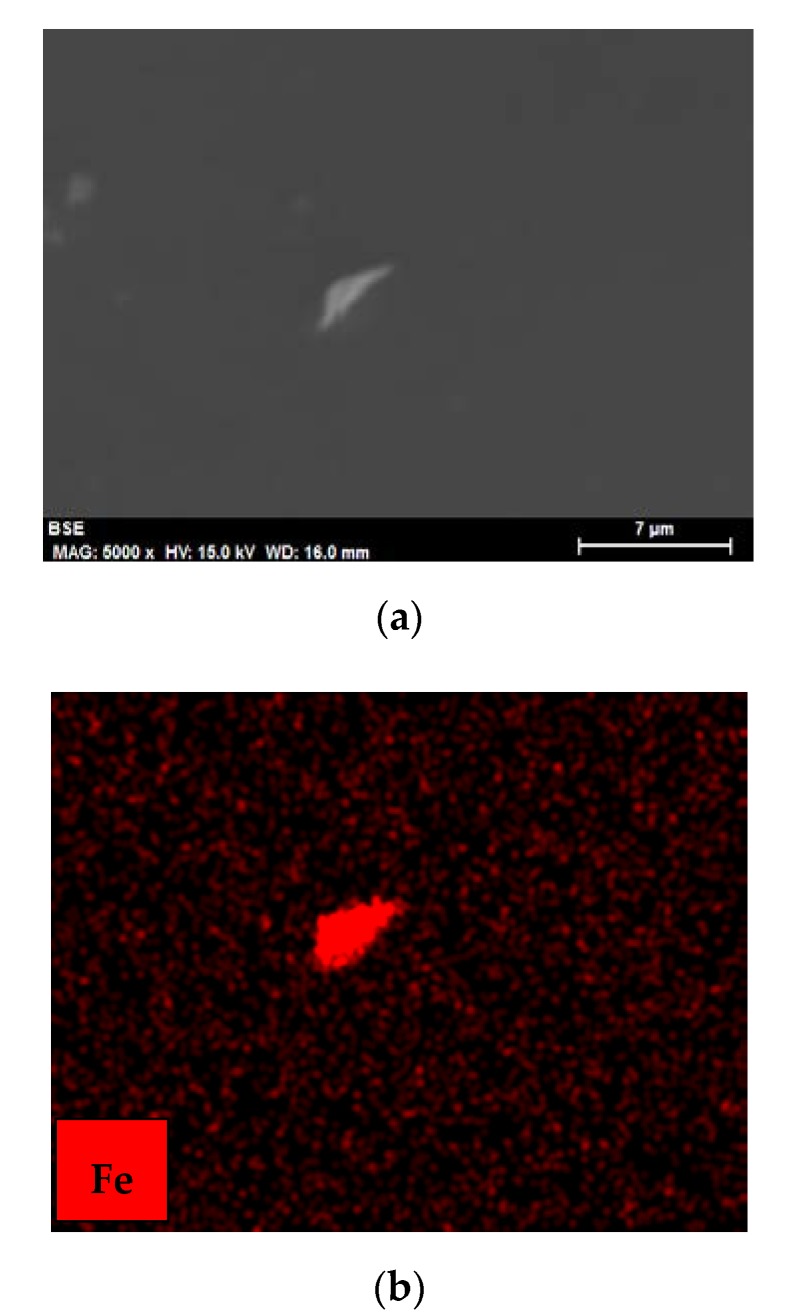
(**a**) Backscattered SEM micrograph of wear debris after lubrication with IL; (**b**) Fe element map.

Wear scars were characterized by surface topography (Figure 5 and Figure 6), cross section profiles (Figure 7) and electron microscopy (SEM) and energy dispersive analysis (EDX) (Figure 8, Figure 9 and Figure 10).

These observations confirmed the order of wear rates (IL > IL+G > IL thin film > (IL + G) thin film) given in Table 2. The best lubrication performance was thus obtained in the presence of graphene, combined with the lubricating film reduction achieved by the spin coating technique.

Figure 5 compares profilometry images of the wear track on stainless steel after lubrication with IL (Figure 5a) and with IL thin film (Figure 5b). The surface damage in this later case was very mild.

When (IL + G) was used (Figure 6a), the wear track was clearly visible, while no surface damage was observed in the profilometry image (Figure 6b) of the steel surface after lubrication with (IL + G) thin film. This observation was in agreement with the formation of a protective surface layer, as has been proposed in previous works [11,12,13,14,19,20,21,22,23,24,30,31].

Cross section profiles of the wear tracks after lubrication with the four lubricants studied (Figure 7) show that neat thick film IL lubricant was not able to protect the stainless steel surface from some degree of plastic deformation and volume loss.

The wide surface scar seen in the SEM micrograph (Figure 8a) also presented some abrasion marks parallel to the sliding direction. This large plastic deformation and abrasion marks were reduced when IL thin film (Figure 8b) or (IL + G) (Figure 8c) were used. Finally, negligible surface damage was obtained when (IL + G) thin film was used (Figure 7 and Figure 8d), where only polishing marks were seen and no plastic deformation nor abrasion marks parallel to sliding were present on the sliding path.

The slightly darker appearance of the sliding path in the central region of Figure 8d could be due to a graphene surface layer. However, it was too thin and narrow to show any carbon percentage increase in EDX and XPS analysis.

In order to further quantify surface changes for each lubricant, surface roughness measurements were carried out inside and outside each wear path. Table 3 shows that outside the sliding paths, all disks showed similar Ra values, while roughness changes were observed inside the load-carrying sliding paths, where surface interactions took place. The order of roughness values were IL > (IL + G) > IL thin film > (IL + G) thin film.

For IL and (IL + G) thick film lubricants, Ra values were one order of magnitude higher inside the wear track, while IL thin film and (IL + G) thin film lubricants maintained roughness values of the same order of magnitude inside and outside the sliding path. The lower roughness increase was obtained for (IL + G) thin film, in agreement with the above discussed surface topography images (Figure 5, Figure 6 and Figure 7) and SEM micrographs (Figure 8).

XPS analysis of the steel disks surface after lubrication with neat IL (Figure 9a) showed that C1s and N1s binding energies were similar inside and outside the wear track (Table 4; Figure 9b–g).

C1s peaks (Figure 9b,c) at 285 eV corresponded to aliphatic carbon; peaks at 286.5 and 286.9 eV were assigned to aliphatic carbon bonded to imidazolium ring; peaks at 288.7 and 288.8 could be due to C1s of the imidazolium ring; and the small atomic percentage peaks at 293.0 and 293.2 eV corresponded to C1s binding energies of the –CF_3_ groups in the [TFSI] anion [32].

N1s binding energies (Figure 9d,e) at 398.2–398.9 eV and at 400.0–400.3 eV were assignable to the imide nitrogen atom of the [TFSI] anion and to the nitrogen atoms in the imidazolium ring of the [OMIM] cation, respectively (Figure 9a).

The most significant difference corresponded to the oxygen peaks (Table 4; Figure 9f,g). O1s binding energies assignable to metal oxides and hydroxides present at the stainless steel surface were observed both outside (Figure 9f), at 530.1 and 531.5 eV, and inside (Figure 9g), at 530.0 and 531.7 eV, the wear track. The atomic percentages (Table 4) of these O1s peaks (blue and red lines in Figure 9f,g) were reduced inside the wear path. The very weak O1s peak outside the wear track at 533.1 eV (green line in Figure 9f) could be attributed to adsorbed water. A new O1s peak at 532.3 eV (green line in Figure 9g) appeared inside the wear path. This new O1s binding energy could be assigned to oxygen atoms in the [TFSI] anion [32] of the IL lubricant. This could be tentatively attributed to the different interaction with the steel surface under load with respect to the load-free outer surface.

Wear debris were only observed in the case of the most severe wear for lubrication with IL (Figure 10a), where the element map (Figure 10b) showed that the particles were composed of iron from the stainless steel disk.

**Figure 11:**
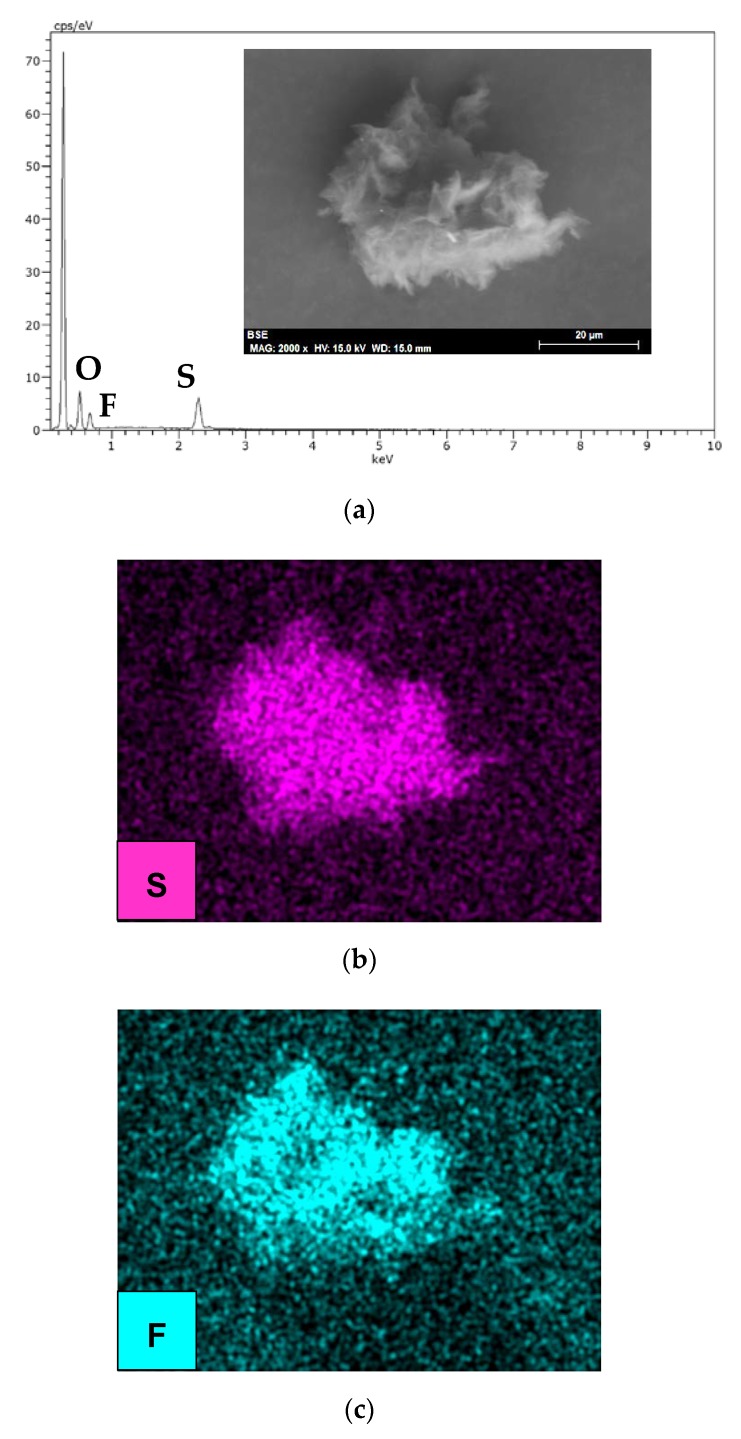
(**a**) SEM micrograph and EDX spectrum of wear debris after lubrication with IL+G, and element maps for (**b**) sulfur and (**c**) fluorine.

After lubrication with the (IL + G) thick film dispersion, graphene particles (Figure 11a–c) recovered from the lubricant after centrifugation and elimination of IL showed that they were composed mainly of carbon (Figure 11a), but also contained sulfur (Figure 11b) and fluorine (Figure 11c) from the [TFSI] anion present in IL.

**Figure 12:**
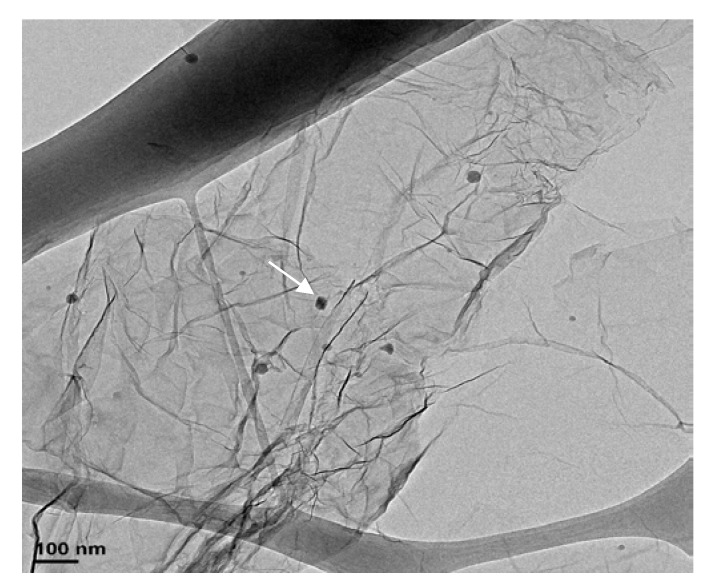
TEM micrograph of wear debris from lubrication with (IL + G) thick film lubricant (after sonication in ethanol and drying). Arrow points to wear debris.

Wear debris from (IL + G) lubricant (Figure 11) were dispersed by sonication in ethanol and dried prior to TEM observation (Figure 12). The original graphene nanolayers were maintained with some nanometric wear debris particles (see arrow in Figure 12) adhered to them. A similar mechanism was previously observed for a thin film dispersion of graphene in a protic ionic liquid [31].

The absence of wear after lubrication with (IL + G) thin film lubricant (Figure 6b, Figure 7 and Figure 8d), was in agreement with the absence of wear debris observation by SEM or TEM.

**Figure 13:**
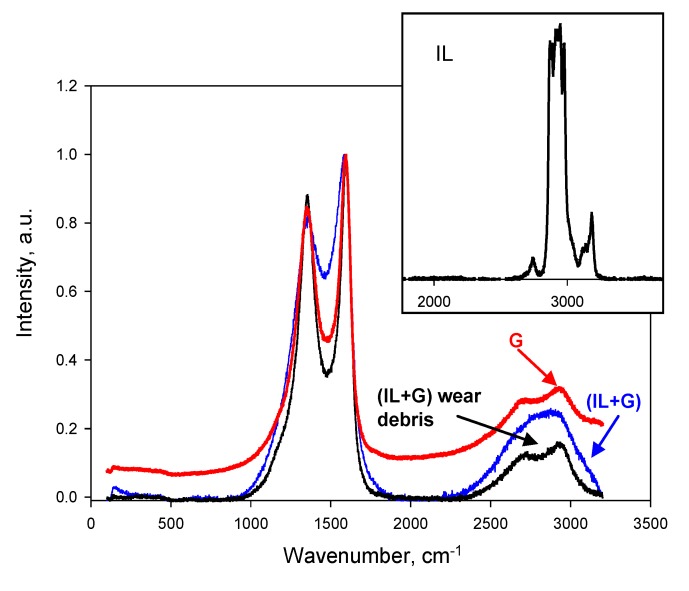
Raman spectra of graphene (G); (IL + G) dispersion and wear debris after lubrication with (IL + G). Inset: Raman spectrum of IL in the 2000–3000 cm^−1^ region.

Figure 13 compares Raman spectra of neat graphene, (IL + G) dispersion before tribological tests and wear debris after lubrication with (IL + G). In the spectrum of (IL + G), the intensity of the band in the 2G region of graphene increased due to the presence of IL molecules, which showed the most intense Raman absorptions due to –C–H, in the same region, as can be observed in the inset in Figure 12.

The spectrum of (IL + G) wear debris shows some changes with respect to that of neat graphene (G). Both show a G band at 1595.0 cm^−1^; however, the D band for (IL + G) wear debris is shifted to a higher value (1354.9 cm^−1^) with respect to graphene (1348.7 cm^−1^), that is, a slight narrowing of the D–G bandwidth took place. A slight increase in I_D_/I_G_ intensity relationship, from 0.85 for neat graphene to 0.88 for wear debris, was observed. These changes could be attributed to compressive stress [33,34] as a consequence of tribological tests.

## 4. Conclusions

(1) A new dispersion of graphene in the hydrophobic ionic liquid 1-octyl-3-methylimidazolium bis(trifluoromethanesulfonyl) imide was obtained and its non-Newtonian behavior was characterized. The viscosity of the new dispersion decreases with increasing temperatures, but it is maintained from 100 to 150 °C.
(2) The tribological performance of the neat ionic liquid and of the dispersion of graphene in ionic liquid was compared with that of thin films of the same lubricants deposited by spin coating on the stainless steel surface. All lubricants present constant friction coefficients along the tribological tests.
(3) The best friction-reducing ability (40% with respect to the rest of lubricants) is obtained for the new spin-coated graphene dispersion in ionic liquid, by the combination of addition of graphene and film thickness reduction.
(4) Wear rate of stainless steel is reduced up to 70% by ionic liquid film thickness reduction, and up to 54% by dispersion of graphene in thick film lubrication.
(5) Surface damage and materials loss are only completely prevented by the combination of both factors, addition of graphene and thickness reduction, in the thin film spin coated dispersion of graphene in ionic liquid. This outstanding anti-wear performance is attributed to the sliding of ionic liquid modified graphene sheets, and to the absence of tribocorrosion at the interface.

## Figures and Tables

**Table 1:** Experimental conditions for pin-on-disk tests.

Parameter	Value
AISI 316L disk thickness (mm)	2.5
AISI 316L disk diameter (mm)	25
AISI 316L surface roughness (Ra; μm)	<0.15
Normal load (N)	0.5
Maximum contact pressure (GPa)	1.56
Mean contact pressure (GPa)	1.04
Sliding speed (m·s^−1^)	0.01
Sliding distance (m)	500
Sliding radius (mm)	9
Sapphire ball sphere radius (mm)	0.75
Lubricant volume (mL)	0.2
Temperature (°C)	23 ± 1
Relative humidity (%)	55 ± 5

**Table 2:** Coefficients of friction and wear rates.

Lubricant	Coefficient of Friction	Wear Rate (mm^3^/N·m)
IL	0.10 (±0.009)	4.1 × 10^−6^ (±4.37 × 10^−7^)
IL + G	0.10 (±0.009)	2.2 × 10^−6^ (±1.53 × 10^−7^)
IL thin film	0.10 (±0.009)	1.2 × 10^−6^ (±4.48 × 10^−8^)
(IL + G) thin film	0.06 (±0.006)	Non measurable

**Table 3:** Average roughness (Ra) values for AISI 316L disks surface after the tribological tests.

Lubricant	Ra (Inside the Sliding Paths; μm)	Ra (Outside the Sliding Paths; μm)
IL	1.47 × 10^−1^ (±1.6 × 10^−2^)	5.79 × 10^−2^ (±5.2 × 10^−3^)
IL + G	1.29 × 10^−1^ (±1.6 × 10^−2^)	5.50 × 10^−2^ (±4.8 × 10^−3^)
IL thin film	8.79 × 10^−2^ (±4.7 × 10^−3^)	4.88 × 10^−2^ (±1.3 × 10^−3^)
(IL + G) thin film	7.75 × 10^−2^ (±9.5 × 10^−3^)	5.33 × 10^−2^ (±4.4 × 10^−3^)

**Table 4:** XPS binding energies and atomic percentages inside and outside the wear track after lubrication with neat IL thick film.

Element	Outside		Inside	
	Binding Energy (eV)	Atomic %	Binding Energy (eV)	Atomic %
C1s	285.0	27.9	285.0	37.9
	286.5	4.5	286.9	4.2
	288.7	6.4	288.8	5.1
	293.0	0.7	293.2	1.0
O1s	530.0	20.1	530.1	17.4
	531.7	25.6	531.5	10.3
			532.3	12.5
	533.1	2.1		
N1s	398.2	0.3	398.9	0.5
	400.0	0.8	400.3	0.7
